# Inhibitory activity of extracts of Hebridean brown seaweeds on lipase activity

**DOI:** 10.1007/s10811-015-0619-0

**Published:** 2015-05-26

**Authors:** Peter Ian Chater, Mathew Wilcox, Paul Cherry, Andrew Herford, Suraiami Mustar, Hannah Wheater, Iain Brownlee, Chris Seal, Jeffrey Pearson

**Affiliations:** Institute for Cell and Molecular Biosciences (ICaMB), Medical School, Newcastle University, Catherine Cookson Building, Framlington Place, Newcastle upon Tyne, NE2 4HH UK; Human Nutrition Research Centre, School of Agriculture, Food and Rural Development, Agriculture Building, Kings Road, Newcastle upon Tyne, NE1 7RU UK; Nanyang Polytechnic, Food and Human Nutrition Department, Newcastle University Office, 180 Ang Mo Kio, Avenue 8, 569830 Singapore, Singapore

**Keywords:** Lipase, Obesity, Fat digestion, Inhibition, Alginate, Polyphenol, *Ascophyllum nodosum*, *Fucus vesiculosus*, *Pelvetia canaliculata*

## Abstract

The effect of three Hebridean brown seaweeds on lipase activity was assessed using a turbidimetric lipase activity assay and an in vitro simulation of the upper digestive tract. The preparations of *Ascophyllum nodosum*, *Fucus vesiculosus*, and *Pelvetia canaliculata* were tested; whole seaweed homogenate, sodium carbonate extract, and ethanol extracts (pellet and supernatant were tested separately). All extracts showed significant inhibition of lipase, suggesting multiple bioactive agents, potentially including alginates, fucoidans, and polyphenols. Whole homogenate extract of *F. vesiculosus* was the most potent inhibitor of Lipase (IC_50_ = 0.119 mg mL-1), followed by ethanol supernatant (IC_50_ = 0.159 mg mL-1) while ethanol pellet and sodium carbonate extract showed relatively weaker inhibition (IC_50_ = 0.360 mg mL-1 and IC_50_ = 0.969 mg mL-1 respectively). For *A. nodosum* and *P. canaliculata*, strongest inhibition occurred with ethanol pellet (IC_50_ = 0.238 and 0.228 mg mL^−1^, respectively). These inhibitory effects were validated in a model gut system. The data presented herein suggests the use of seaweed as a potential weight management tool is deserving of further investigation.

## Introduction

Excess fat storage and consumption of high energy and high fat diets are associated with an array of health complications including increased morbidity (Bellanger and Bray 2005) and comorbidities including cardiovascular disease (Logue et al. 2011; Nanchahal et al. 2005), Type 2 diabetes (Garg et al. 2014), cancers of the gastrointestinal tract and kidney (Calle et al. 2003; Fujihara et al. 2012; Garg et al. 2014), metabolic syndrome (Fujihara et al. 2012), and osteoarthritis (Tabassum and Batty 2013). Finding effective treatments and interventions for obesity and associated diseases is therefore paramount.

The inhibition of the digestion and absorption of dietary fat has been demonstrated to be an effective treatment for obesity, as seen with the pancreatic lipase inhibitor Orlistat (Lucas and Kaplan-Machlis 2001). However, side effects including steatorrhea and incontinence can make it an unpleasant treatment for the patient and reduce compliance (Padwal and Majumdar 2007). There is therefore a drive to discover and characterise novel lipase inhibitors with reduced side effects (Balasubramaniam et al. 2013).

Brown seaweeds (Phaeophyta) are a rich source of bioactive polysaccharides (including alginate, fucoidan, and laminarin) and polyphenols (including phenolic acids, flavonoids, stilbenes, and lignans (O’Sullivan et al. 2010; Kim et al. 2014). There is evidence to suggest that these bioactive compounds may be potential modulators of enzyme activity. Alginate has been shown to inhibit pepsin (Rogalska et al. 1990) and pancreatic lipase (Wilcox et al. 2014) in vitro, where inhibition is dependent upon the frequency of mannuronate and guluronate residues in the binary copolymer (Wilcox et al. 2014). A number of studies have demonstrated in vitro inhibition of α-amylase and α-glucosidase by extracts of the brown seaweeds *Ascophyllum nodosum* and *Fucus vesiculosus* (Nwosu et al. 2011; Roy et al. 2011; Zhang et al. 2006a; Apostolidis and Lee 2010). Furthermore, polyphenol rich plant extracts from tea and soft fruits (e.g., blueberries and raspberries) have demonstrated inhibition of pancreatic lipase (Sergent et al. 2012). Brown seaweeds including the species *A. nodosum*, *F. vesiculosus* and *Pelvetia canaliculata* have demonstrated high antioxidant activity (Wang et al. 2009; O’Sullivana et al. 2011; Nwosu et al. 2011; Apostolidis and Lee 2010) which is attributed to their high polyphenol content.

Edible seaweeds (macroalgae) are common components of diets throughout the world (Brownlee et al. 2012). In the UK, seaweeds are seen as an underutilised resource despite being abundantly available, sustainably harvested, and already in use in the UK food industry. The brown seaweeds studied herein, *A. nodosum*, *F. vesiculosus*, and *P. canaliculata*, are predominantly found on the coastlines of Ireland, Scotland, Norway, Iceland, and Canada and are used by UK food producers as a salt replacer. The aim of this paper is therefore to investigate the effects of these three species of Hebridean seaweed on lipase activity. Whole seaweed and crude seaweed extracts are tested in an in vitro system and in an artificial model gut system to assess their potential to inhibit fat digestion in the diet.

## Materials and methods

All reagents (unless otherwise stated) were purchased from Sigma-Aldrich (USA). The three species of seaweeds were provided in dried, powdered form by Seagreens Ltd. (Handford Way, West Sussex, UK). *A. nodosum*, *P. canaliculata*, and *F. vesiculosus* are brown seaweeds which are currently being harvested for human consumption in the Outer Hebrides on the west coast of Scotland. To preserve the quality of the seaweed, the drying process is completed at a low temperature, within 12–24 h of being cut.

### Sample preparation

Four extractions of each seaweed were prepared from dried powder as described in Table 1.

**Table 1:** The four extraction methods used to isolate bioactive compounds from seaweeds

Seaweed fraction	Method
Seaweed homogenate	Samples were homogenised in the protocol buffer for 30 min at the required concentration (5 mg mL^−1^) and filtered through glass wool before use.
Ethanol extraction—Supernatant	Alcohol extracts were prepared by homogenising the sample in ethanol at 100 mg mL^−1^ for 30 min. The sample was centrifuged at 3000 rpm for 10 min at 20 °C. The supernatant was then poured off and evaporated down to give a supernatant solid.
Ethanol extraction—Pellet	Alcohol extracts were prepared by homogenising the sample in ethanol at 100 mg mL^−1^ for 30 min. The sample was centrifuged at 3000 rpm for 10 min at 20 °C. The supernatant was then poured off and the pellet dried to give a fine powder.
Sodium carbonate extract	3 g of dried seaweed sample was mixed overnight in 0.2 M HCl. Ninety milliliters of deionised water (dH_2_O) were added to the sediment (the compounds insoluble in acid) and left to mix overnight. This solution was centrifuged at 5000 rpm for 5 min at 4 °C. Sixty milliliters 1 M Na_2_CO_3_ were added to the pellet and stirred at 70 °C for 3 h. The solution was filtered through glass wool overnight. The filtered solution was then centrifuged at 10,000 rpm for 20 min at 4 °C. The supernatant was exhaustively dialyzed at 4 °C and freeze dried to give a white, spongy, and fibrous extract.

### Pancreatic lipase inhibition assay

Lipase activity was assayed using a turbidimetric method with minor modifications (Balasubramaniam et al. 2013; Vogel and Zieve 1963). Buffer diluent was made up of 0.033 M citric acid, 0.343 M KOH, 0.033 M H_3_PO_4_, 0.033 M H_3_BO_3_, and 0.35 % bile acid (Taurodeoxycholate or Deoxycholic acid Na salt) adjusted to a pH of 7.5 using HCl. Substrate solutions containing seaweed extracts were made up of 5 mg mL^−1^ seaweed and re-homogenised for a further 2 min, then diluted using substrate solution to the following concentrations: 5, 2.5, 1.25, 0.5, 0.25, 0.125, 0.05, 0.0125, 0.005, and 0.0025 mg mL^−1^. Sample dose concentrations were based on the previous investigations of similar compounds (Balasubramaniam et al. 2013; Wilcox et al. 2014). The substrate solution was used within 6 h of preparation. The enzyme solution contained 1 mg mL^−1^ lipase and 15 μg mL^−1^ co-lipase in buffer diluent. Samples were incubated at 37 °C for 10 min and 200 μL of substrate solution was transferred to 10 μL lipase or buffer solution. Eight readings (5 min intervals) using a BioTek EL808 96-well plate spectrophotometer were taken at 405 nm (37 °C) Tables 2 and 3.

**Table 2:** IC_50_ values for extract preparations of the seaweeds

Extract	IC_50_ (mg mL^−1^)		
	*Ascophyllum nodosum*	*Fucus vesiculosus*	*Pelvetia canaliculata*
Homogenate	0.748	0.119	0.379
Ethanol pellet	0.238	0.360	0.228
Ethanol supernatant	1.932	0.159	1.822
Sodium carbonate	2.098	0.969	0.789

**Table 3:** Total Polyphenol content released from seaweed extracts during enzymatic digestion at 10 and 70 min (mg GAE g^−1^ dry weight) All values are means of triplicate assays ± SE.

Seaweed source	In vitro digestion stage	
	10 min	70 min
*Ascophyllum nodosum*	11.0 ± 0.5^a^	34.9 ± 0.5^b^
*Fucus vesiculosus*	28.9 ± 0.4^e^	61.3 ± 1.9^f^
*Pelvetia canaliculata*	19.6 ± 0.3^c^	53.2 ± 0.7^d^

At every time-point, significant differences were shown between all samples as indicated in table (different superscript letters indicate statistical difference)

Porcine pancreatic lipase (at a final concentration of 0.048 mg mL^−1^) was used as a control and represents 100 % lipase activity, and inhibition is shown as a percentage of control lipase activity. The pancreatic lipase inhibitor Orlistat (at a final concentration of 0.24 mg mL^−1^, based on the previous investigations (Wilcox et al. 2014)) was used as a positive control giving 100 % inhibition.

The change in absorbance from time point zero was calculated for each substrate solution:

$$ \varDelta\ \mathrm{Absorbance} = \mathrm{Absorbance}\ {T}_0\ \hbox{--}\ \mathrm{Absorbance}\ {T}_x $$

Percentage of lipase activity:

$$ \%\kern0.75em \mathrm{activity}\ \mathrm{relative}\ \mathrm{t}\mathrm{o}\ \mathrm{t}\mathrm{he}\ \mathrm{substrate}\ \mathrm{control}\ \mathrm{at}\kern0.5em {T}_x = \frac{\varDelta\ \mathrm{Absorbance}\ \mathrm{sample}\ }{\varDelta\ \mathrm{Absorbance}\ \mathrm{control}} \times 100 $$

### Synthetic gut simulation

Digestion of an olive oil substrate was assayed in a model gut system. The system was set up as described in Houghton et al. (2014), and samples were collected and precipitated in 10 % trichloroacetic acid to stop enzyme activity and fat digestion was assayed using the Zenbio Glycerol Analysis kit as per instructions (Zenbio, Durham, USA) (Houghton et al. 2014).

#### Procedure

*Salivary Phase*—At *t* = −10, samples and controls were prepared in 5 mL artificial saliva and added to 5 mL dH_2_O under stirring for 10 min on rollers. *Gastric Phase*—At *t* = 0, salivary preparations were added to a resting reservoir of 50 mL synthetic gastric juice (pre-incubated to 37 °C). The remaining gastric juice was added via a Watson Marlow peristaltic pump (rate = 0.5 mL min^−1^). Gastric diluent is prepared prior to assay at *t* = 20 to avoid pepsin autodigestion. *Pancreatic Phase*—25 mL porcine bile  was added at *t* = 60 and the pumping of synthetic gastric juice was stopped, filtered synthetic pancreatic juice was then pumped in at a rate of 0.5 mL^−1^. The small-intestinal phase was continued until *t* = 180. Samples of 1 mL were taken at *t* = 0, 5, 10, 15, 30, 45, 60, 60b, 65, 70, 75, 90, 105, 120, 150, and 180. *t* = 60b represents a second sample at *t* = 60 after the addition of fresh porcine bile; the removal of sample volume was corrected for in calculations. Samples were immediately mixed 2:1 with 10 % *w/v* trichloroacetic acid to stop enzyme activity and stored at 4 °C overnight to allow for precipitation, centrifuged at 10,000 r.p.m. for 10 min at room temperature, and supernatant was then analysed (Alfred and Rao 1971; Mossner et al. 1989; Montalto and Bensadoun 1993).

#### Glycerol analysis

Fat digestion was assayed by quantifying free glycerol released from 1 g of olive oil substrate using the Zenbio Glycerol Analysis Kit (Zenbio, USA) (Zenbio 2010). A glycerol standard curve was prepared. Five microliters of each sample were plated out, and 40 μL of glycerol reagent A was added to all wells and incubated for 30 min. Absorbance was measured at 540 nm. A background control was prepared with no substrate, and extract controls were prepared with extract and no substrate.

### Assessing the polyphenol content

An in vitro digestion procedure was based on a previous method developed by (Aura et al. 1999). The total phenolic content (TPC) of the extracts was determined using Folin-Ciocalteu reagent following a previous method (Zhang et al. 2006b). The assay was carried out on a 96-well microplate using gallic acid as the standard. Phenolic levels are expressed as a gallic acid equivalent, which is used as a common reference compound (Pourmorad et al. 2006).

## Results

### Pancreatic lipase inhibition assay

**Fig. 1:**
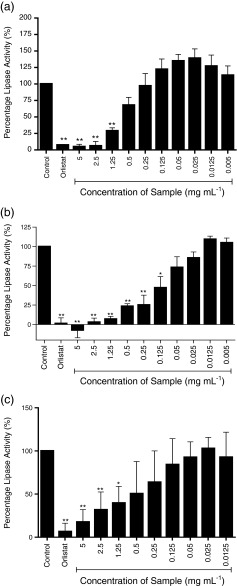
**a**–**c** Inhibition of pancreatic lipase activity by whole seaweed homogenate of three seaweeds; **a** *A. nodosum*, **b** *F. vesiculosus*, and **c** *P. canaliculata.* Data are represented as a percentage change in turbidity relative to the positive control (100 %). The data represent mean values ± SD of at least *n* = 4. *p < 0.05, **p < 0.001

**Fig. 2:**
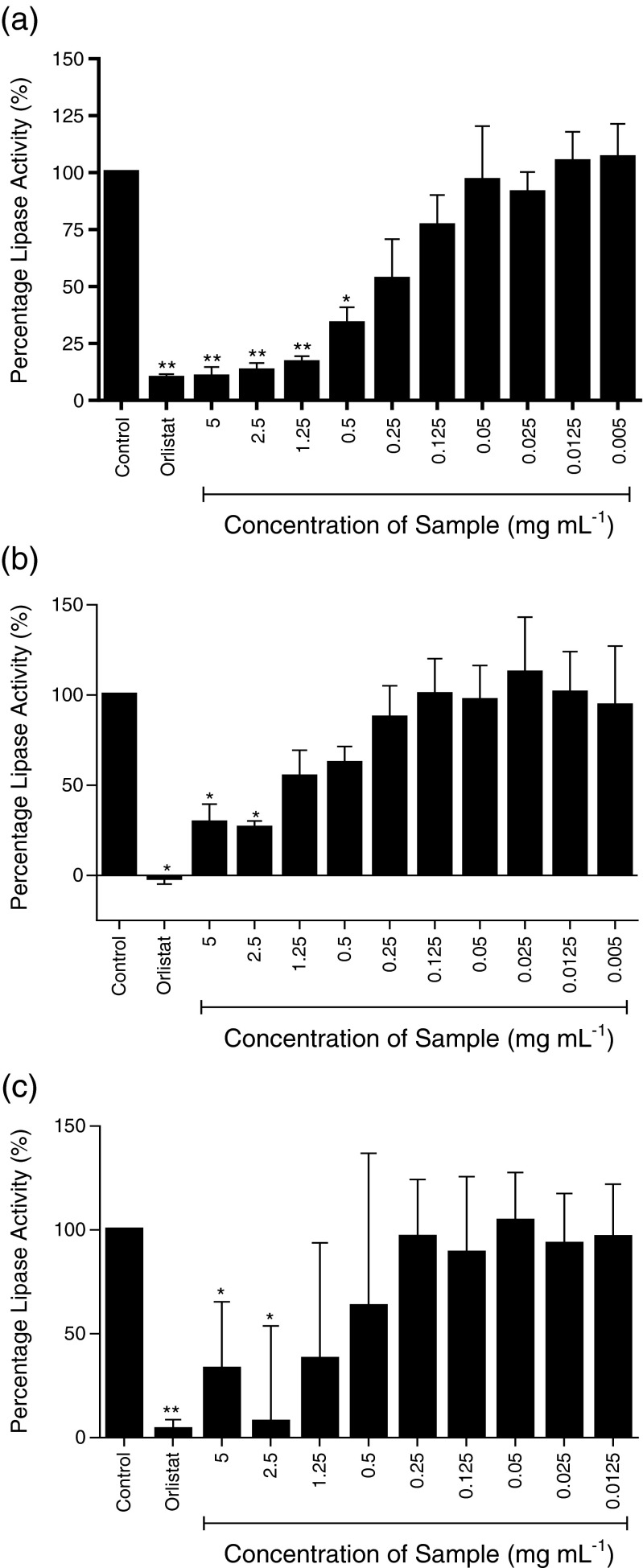
**a**–**c** Inhibition of pancreatic lipase activity by ethanol extract—pellet preparation of three seaweeds; **a** *A. nodosum*, **b** *F. vesiculosus*, and **c** *P. canaliculata*. Data are represented as a percentage change in turbidity relative to the positive control (100 %). The data represent mean values ± SD of at least *n* = 4. *p < 0.05, **p < 0.001

**Fig. 3:**
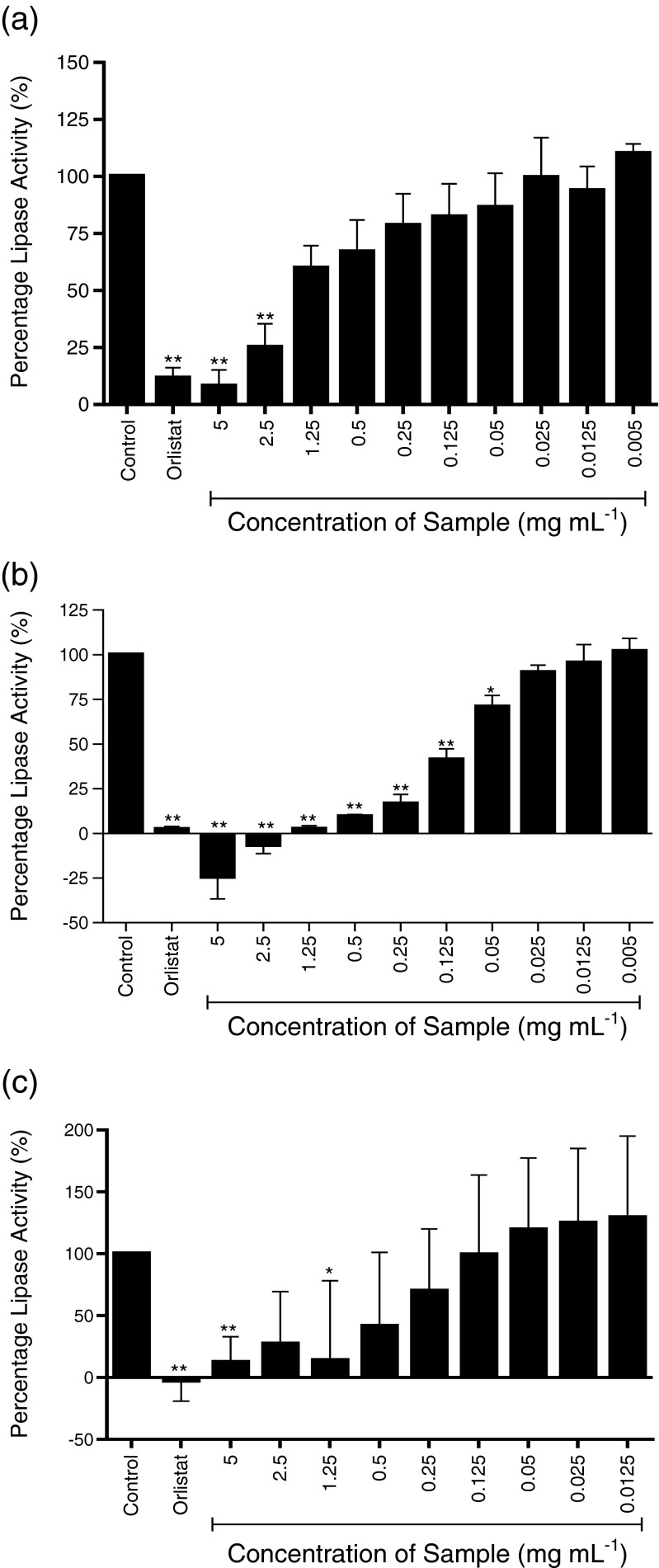
**a**–**c** Inhibition of pancreatic lipase activity by ethanol extract—supernatent of three seaweeds; **a** A*. nodosum*, **b** *F. vesiculosus*, and **c** *P. canaliculata*. Data are represented as a percentage change in turbidity relative to the positive control (100 %). The data represent mean values ± SD of at least *n* = 4. *p < 0.05, **p < 0.001

**Fig. 4:**
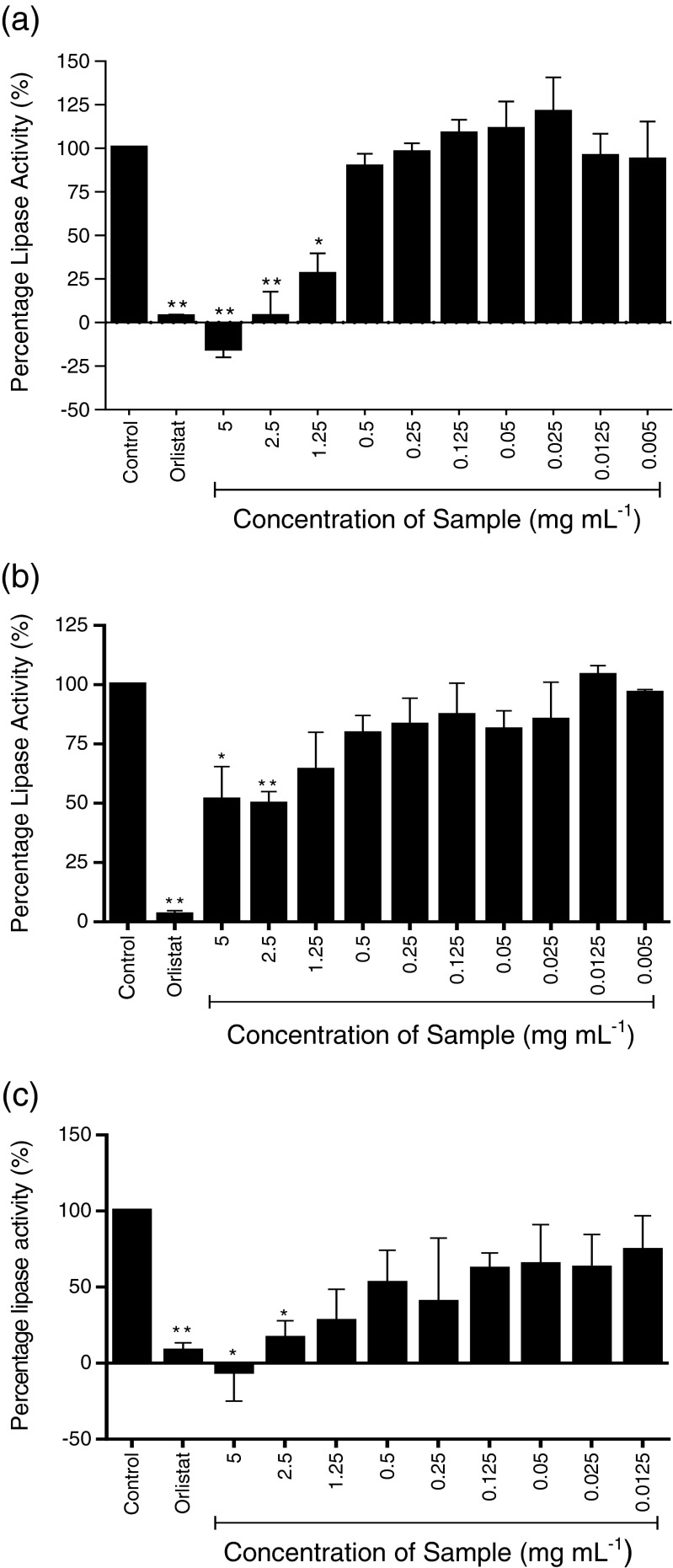
**a**–**c** Inhibition of pancreatic lipase activity by alkaline extraction of three seaweeds; **a** *A. nodosum*, **b** *F. vesiculosus*, and **c** *P. canaliculata*. Data are represented as a percentage change in turbidity relative to the positive control (100 %). The data represent mean values ± SD of at least *n* = 4. *p < 0.05, **p < 0.0001

Three species of seaweed (a) *A. nodosum*, (b) *F. vesiculosus*, and (c) *P. canaliculata* were tested and four preparations of seaweed were used; whole seaweed homogenate (Fig. 1a–c), ethanol extraction—pellet (Fig. 2a–c), ethanol extraction—supernatant (Fig. 3a–c), and sodium carbonate extract (Fig. 4a–c).

Figure 1a shows the dose response inhibition of lipase activity by *A. nodosum* homogenate. Significant inhibitions (*p* < 0.001) are observed at 5, 2.5, and 1.25 mg mL^−1^. At 5 and 2.5 mg mL^−1^, lipase activity was reduced to less than 10 % of control, comparable to that seen with the Orlistat positive inhibition control. A significant inhibition of 68 % was observed at 1.25 mg mL^−1^ (*p* < 0.001). The IC_50_ of the *A. nodosum* homogenate was 0.748 mg mL^−1^, indicating that 0.748 mg mL^−1^ of *A. nodosum* homogenate was required to reduce the activity of 0.048 mg mL^−1^ lipase by half. Non-significant activations were observed at concentrations of 0.125 mg mL^−1^ and below.

Figure 1b shows near complete inhibition of lipase activity by *F. vesiculosus* homogenate at 5 mg mL^−1^ (*p* < 0.001). At 1.25 and 2.5 mg mL^−1^ extracts reduced activity to below 10 % of control (*p* < 0.001), 75 % inhibition was observed at 0.25 mg mL^−1^ (*p* < 0.0001), and at 0.125 mg mL^−1^ activity was reduced to below 50 % of control (*p* < 0.001). The IC_50_ of the homogenate was 0.119 mg mL^−1^, making it the most potent of all the seaweed extracts tested (Table 3).

Figure 1c shows inhibition of lipase activity by *P. canaliculata* homogenate at 5, 2.5, and 1.25 mg mL^−1^ of 82, 68, and 60 %, respectively, compared with control. The IC_50_ of the *Pelvetia* homogenate was 0.379 mg mL^−1^ (Table 3).

The ethanol extraction pellet comprises of all of the components of seaweed insoluble in ethanol, and with all three seaweed samples, significant inhibition was observed, with IC_50_ values of 0.228, 0.238, and 0.360 mg mL^−1^ for *P. canaliculata*, *A. nodosum*, and *F. vesiculosus*, respectively (*p* < 0.05)*.*

The pellet remaining after ethanol extraction  of *A. nodosum* achieved significant inhibition of greater than 75 % at 5, 2.5, and 1.25 mg mL^−1^ (*p* < 0.05) (Fig. 2a). The pellet extracts of *F. vesiculosus* and *P. canaliculata* achieved inhibitions higher than 60 % only at 5 and 2.5 mg mL^−1^ (Fig.2b, c), yet all three had similar dose response profiles and therefore similar IC_50_ values.

According to the IC_50_ values calculated, the whole seaweed homogenate of *F. vesiculosus* was more potent than the ethanol extract pellet. Across the three seaweed samples tested, the ethanol extract pellets of *P. canaliculata* and *A. nodosum* were the most potent extracts of those seaweeds confirming that the ethanol insoluble fraction of the seaweeds has significant anti-lipase activity (*p* < 0.05).

The ethanol supernatant of all three seaweeds showed potent inhibition of lipase activity at the higher concentrations (Fig. 3). At 5 mg mL^−1^, *F. vesiculosus* extract completely inhibited lipase activity, and at the same concentration, *A. nodosum* and *P. canaliculata* showed inhibition of lipase greater than 80 % of control. *A. nodosum* and *P. canaliculata* extracts showed similar inhibition profiles, with IC_50_ values of 1.932 and 1.822 mg mL^−1^, respectively. The ethanol extract of *F. vesiculosus*, however, demonstrated much more potent inhibition of lipase, with significant inhibition observed at all tested concentrations between 0.05 and 5 mg mL^−1^ and an IC_50_ value of 0.159, an order of magnitude lower than the other two seaweed samples.

The sodium carbonate extracts of the seaweeds showed significant inhibition of lipase activity (Fig. 4), however, while *P. canaliculata* and *A. nodosum* completely inhibited lipase activity at 5 mg mL^−1^, the sodium carbonate extract of *F. vesiculosus* only yielded a 48 % inhibition. The sodium carbonate extract would be expected to contain mainly alginate, which has previously been shown to be a potent lipase inhibitor.

All of the samples and extracts tested showed a dose responsive inhibitory effect on pancreatic lipase compared with the control, and the IC_50_ values are summarised in Fig. 3.

### Gut-like conditions to assess the inhibitory potential of *F. vesiculosus*

*F. vesiculosus* was selected for testing in a synthetic gastrointestinal simulation as it demonstrated the highest inhibition (with whole homogenate) and the most consistent levels of inhibition across other sample extracts. Homogenate and pellet extracts were tested at 500 mg and supernatant at 250 mg. Sodium carbonate extract was not tested as there were insufficient amounts of raw seaweed to produce enough extract for testing.

**Fig. 5:**
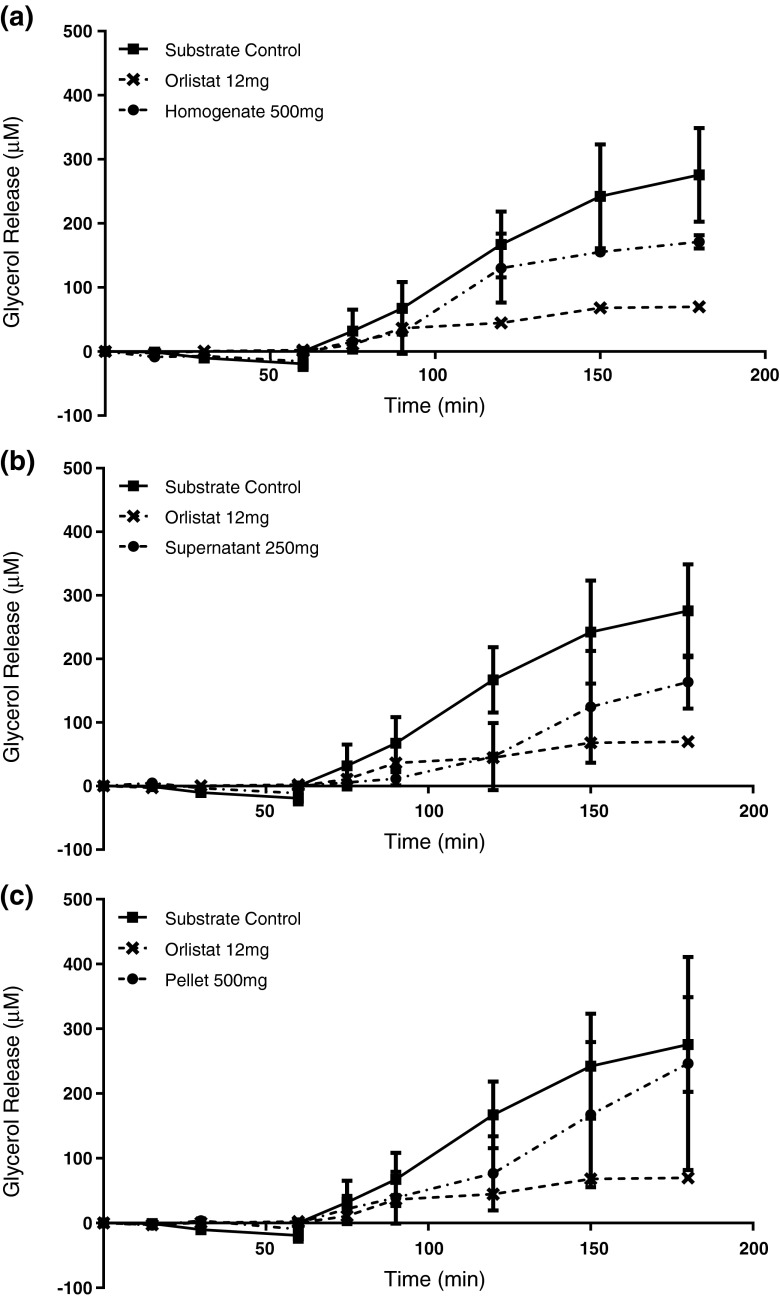
Free glycerol released from olive oil substrate during 180 min of simulated digestion in the presence of *F. vesiculosus* extracts. **a** Homogenate extract at 500 mg, **b** ethanol supernatant fraction at 250 mg, and **c** ethanol pellet fraction at 500 mg. All extracts were tested in parallel with a control sample containing 1 g olive oil substrate + diluents, where *n* = 3 for each sample with SD. Twelve milligram Orlistat was a positive inhibition control. Mean values were analysed using a two way ANOVA with Tukey’s post hoc test

Orlistat significantly reduced fat digestion by 77, 73, and 71 % compared with the substrate control at 120, 150, and 180 min, respectively (*p* < 0.0001) (Fig. 5). Only the homogenate and the ethanol supernatant demonstrated reduced fat digestion in this assay, reducing the production of free glycerol significantly during the small intestinal phase of the model gut system. At 500 mg, the homogenate extract (Fig. 5a) demonstrated a reduction of glycerol release from 120 min onwards. In the presence of 500 mg homogenate, the concentration of glycerol in the model gut solution was 130 μM (*p* < 0.05), 155 μM (*p* < 0.0005), and 171 μM (*p* < 0.05) at 120, 150, and 180 min, respectively, indicating reductions of 33, 38, and 28 % compared with the control.

The ethanol supernatant fraction (Fig. 5b) demonstrated the strongest inhibition when tested in the model gut system. Two hundred and fifty milligrams of the supernatant extract significantly reduced fat digestion from 120 min onwards during the small intestinal phase of digestion. At *t* = 120, the free glycerol concentration was reduced by 76 % compared with the substrate control (46.33 and 190.4 μM for extract and control, respectively; *p* = 0.001). At *t* = 150 min, free glycerol concentration was reduced by 60 % (*p* < 0.0001) compared with the substrate control and at *t* = 180 min by 54 % (*p* < 0.0001). These results indicate that the bioactive compounds in the supernatant fraction hold strong potential as pancreatic lipase inhibitors, this differs from IC_50_ data derived from the plate assay in Fig. 3a–c, where the homogenate was more effective than the supernatant fraction. No significant changes in glycerol release were observed with the 500 mg pellet sample (Fig. 5c).

### Assessment of total polyphenol content

**Fig. 6:**
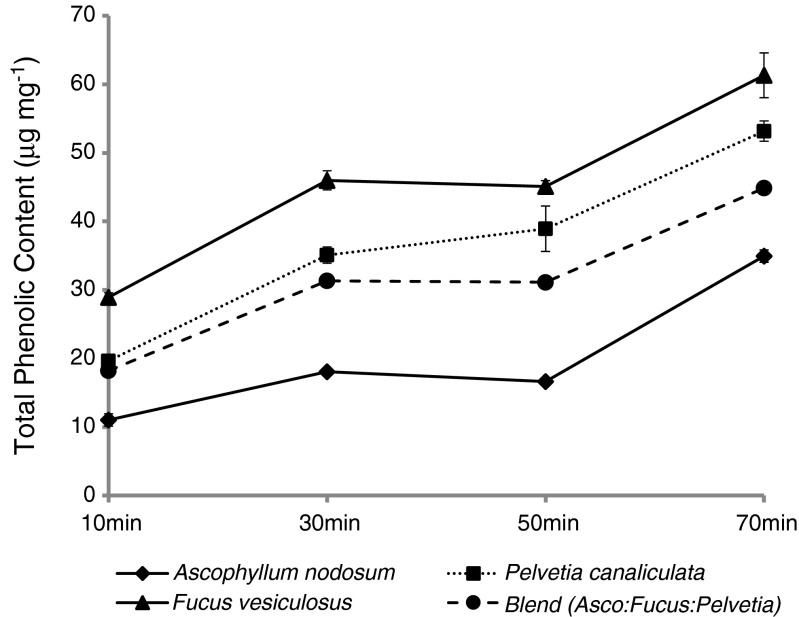
Total polyphenol content released from seaweed extracts during the in vitro enzymatic digestion. All values are means of triplicate assays ± SD

The presence of polyphenols was detected in all of the seaweed samples tested, and the TPC released from the extracts increased over the course of an in vitro digestion procedure (Fig. 6). For all species, there was a significant and substantial increase in TPC release between 10 and 70 min. All values differed significantly from the other species at each stage of the digestion (Table 4). The TPC (mg GAE g^−1^ dry weight) release from extracts during the in vitro enzymatic digestion was in the order of *F. vesiculosus* > *P. canaliculata > A. nodosum*.

**Table 4:** Relationship between TPC and lipase inhibition

Extract	Total phenolic content (TPC) mg^−1^ GAE dry weight after 70 min of in vitro digestion	IC_50_ (mg mL^−1^)			
		Ethanol pellet	Sodium carbonate	Whole Seaweed homogenate	Ethanol supernatant
*Ascophyllum nodosum*	34.9	0.238	2.098	0.748	1.932
*Fucus vesiculosus*	61.3	0.36	0.969	0.119	0.159
*Pelvetia canaliculata*	53.2	0.228	0.789	0.379	1.822

## Discussion

Seaweed extracts contain multiple uncharacterized compounds which have bioactive potential. These compounds may provide diverse health benefits through pharmacological action. Their bioactivity includes acting as an anti-inflammatory, antioxidant, and as a potential prebiotic (O’Sullivan et al. 2010) and seaweed isolates have previously shown inhibition of digestive enzymes (Lordan et al. 2013).

The brown seaweeds investigated in this study, *A. nodosum*, *F. vesiculosus*, and *P. canaliculata*, contain bioactive biopolymers including alginate, fucoidan, and laminarin, as well as non-polysaccharide bioactive compounds such as polyphenols (O’Sullivan et al. 2010; Kim et al. 2014). The abundance of polysaccharides present is dependent on harvesting season (Rioux et al. 2007). This means that seaweed extracts will vary somewhat in composition and produce different yields of polysaccharide.

Four extractions of each seaweed were prepared from dried powder as described in Table 1. The seaweed homogenate contained all compounds present in each of the seaweeds. The ethanol extraction separated the ethanol soluble and insoluble compounds (supernatant and pellet, respectively). The ethanol supernatant would be expected to contain carotenoids, polyphenols such as phlorotannins and the ethanol pellet would be expected to contain the ethanol insoluble carbohydrates including alginate and fucoidan (Warkoyo and Saati 2011; Samee et al. 2009). The sodium carbonate extract is expected to be predominantly alginate (Fenoradosoa et al. 2009). The whole homogenate extract of *F. vesiculosus* was the most potent lipase inhibitor in the lipase activity assay, and supernatant portions of the ethanol extract all showed potent inhibition with the sodium carbonate extract showing weaker inhibition. These data suggest that both ethanol soluble and insoluble components of this seaweed have significant potent inhibitory effect which, in combination in the whole homogenate sample, acts to give an additive effect of increased inhibition. While the homogenate appeared the most potent lipase inhibitor in the lipase activity assay, the supernatant fraction showed the largest reduction in fat digestion in the physiologically relevant gut model. This may be because bioactive compounds present in the homogenate had greater affinity for other digestive enzymes present in the model gut system such as pepsin, amylase, and trypsin, which could potentially reduce the inhibition of lipase by the homogenate in the gut model (Chater 2014). No significant changes were observed with pellet sample, this may be due to large variation in the data or that the amount of pellet used was too small to produce an effect (given that the pellet IC_50_ was larger than the supernatant and homogenate).

For *A. nodosum*, moderate inhibition was observed with the homogenate, strong inhibition with the pellet extract and only weak inhibition with the supernatant and sodium carbonate extracts. This suggests that in this seaweed, non-alginate ethanol insoluble components have potent inhibitory effects on lipase activity. This pattern was also observed for *P. canaliculata*, where the pellet extract displayed strong inhibition, the homogenate moderate inhibition and the supernatant and alkaline fractions lower levels of inhibition.

The mechanism of pancreatic lipase inhibition by these seaweed extracts is unknown, however, the mechanisms of action have been proposed for other pancreatic lipase inhibitors. For example, physical blocking of the interaction between TAG (in oil phase) and the enzyme (in aqueous phase) at the water–oil interface preventing lipase accessing the substrate (Wilcox et al. 2014; Winkler et al. 1990). Binding of substrate and inhibitor, preventing substrate entering the active site of lipase, may be possible. A mechanism similar to that observed for the inhibition of lipase by pectin may occur, where the Ser-Asp-His/Glu catalytic triad of the lipase active site (Winkler et al. 1990; Emmerich et al. 1992; Kumar and Chauhan 2010) undergoes a conformational change after protonation of histidine or serine residues by –COOH groups of pectin (Kumar and Chauhan 2010). As such, the enzyme-substrate complex does not form, protonation of the first glycerol carbon is prevented, and cleavage of the glycerol-fatty acid sn-1 ester bond cannot occur. It is possible that seaweed components including alginate and polyphenols act in a similar manner to inhibit lipase activity. As discussed by Wilcox et al. (2014), carboxyl groups in G-block structures of alginate are similarly oriented to the backbone of pectin molecules and it is therefore possible that they interact with the active site via a similar mechanism (Wilcox et al. 2014). The low inhibition observed in the lipase activity assay for the sodium carbonate extracts in this study (relative to other extracts) may be due to alginate gelation causing reduced interaction between alginate and the enzyme/substrate (De Boisseson et al. 2004). Previous studies indicate that the G:M ratio (unknown in these extracts) is a determining factor for alginate inhibition of pancreatic lipase, where alginate bioactivity is dependent on sugar residue composition and molecular weight (Wilcox et al. 2014). It may therefore be that the alginate extracted from the three species of seaweed has a low G:M ratio. Characterising the G:M ratio of the three seaweed alginates would clarify this observation.

Fucoidan, another fibrous biopolymer present in *F. vesiculosus*, is comprised of sulfated fucose residues alongside other monosaccharides (Li et al. 2008). Fucoidans precipitate in alcohol and were present in the ethanol pellet fraction (and homogenate). It was not possible to detail any inhibition of pancreatic lipase by fucoidan alone because the pellet fraction contained multiple compounds. Despite this, recent evidence suggests that fucoidan from *F. vesiculosus* can inhibit α-glucosidase by 80 % depending upon season of harvest, with seaweed harvested in autumn yielding the highest levels of inhibition (Kim et al. 2014).

Alcohol extracts of *F. vesiculosus* are reported to contain approximately 23 % polyphenols (Zhang et al. 2006b). Polyphenols can be consumed at levels up to 1 g day^−1^ in the diet (Scalbert et al. 2005). The inhibition by the supernatant fraction may be due to its polyphenol content, given their previously demonstrated ability to inhibit digestive enzymes in vitro such as α-amylase and α-glucosidase (Mai et al. 2007; Apostolidis and Lee 2010), where hydroxylation of flavonoids increases α-glucosidase inhibition. Furthermore, it has been demonstrated that polyphenols such as phlorotannin can interact with and precipitate proteins (Stern et al. 1996). There are some limitations to the use of polyphenols therapeutically as they degrade in UV light and at high temperatures (Volf et al. 2014) potentially reducing their utility as a food additive targeted at reducing pancreatic lipase activity. Moreover, polyphenols are a non-fiber compound and would not be able to bind undigested fats, therefore if included in the diet may cause steatorrhea, similar to Orlistat (Hsu et al. 2006).

The TPC of the seaweeds released during in vitro digestion differed significantly between species. *F. vesiculosus* showed the highest polyphenol release ranging from 29 to 61 mg g^−1^ through the in vitro digestion while *A. nodosum* was the lowest at 11–35 mg g^−1^. Apostolidis et al. (2010) found that the TPC of *A. nodosum* extracted with water at 80 °C for 30 min was in the range 22–35 mg g^−1^ when investigating the seasonal variation in TPC of *A. nodosum* harvested in Canada (Apostolidis and Lee 2010). This is similar to the range found in *A. nodosum* in this study. Wang et al. (2009) also reported that the TPC of *F. vesiculosus* was higher than that of *A. nodosum* (Wang et al. 2009).

There are limitations to this assessment of TPC, as the stability of the polyphenols through the in vitro digestion is unknown, and the Folin–Ciocalteu method most likely overestimates TPC because it also reacts with other hydroxyl containing species (this is why polyphenols could not be assessed in the presence of pancreatin). The seaweed *F. vesiculosus* had the highest level of TPC at 70 min and the lowest IC_50_ values for both homogenate and supernatant. *P. canaliculata* was the next most potent inhibitor for both homogenate and supernatant and had the second highest TPC (Table 4). The homogenate and ethanol supernatant extracts would be expected to be rich in polyphenols, but the sodium carbonate extract and ethanol pellet extraction would not.

While these data suggests that extracts rich in polyphenols tend to be more potent inhibitors of lipase, further investigation is required to characterise and purify the polyphenols present in the seaweed samples to confirm a causal relationship. While the polyphenol rich extracts *F. vesiculosus* and *P. canaliculata* tend to inhibit more potently, there is no linear correlation between levels of polyphenols and levels of lipase inhibition. It is therefore possible that the type of polyphenols is also important, and the specific composition of the polyphenols in *F. vesiculosus* is important in their inhibitory effects.

It is clear that with the presence of fucoidans, alginates, polyphenols, and other components, seaweeds are comprised of multiple bioactive agents which may act alone or in concert to be potent inhibitors of lipase activity with potential therapeutic applications. Characterising and isolating the various components of the seaweeds would be useful for further study, while nutritional component analysis would provide information for suitability as a functional food ingredient.

Evidence from the lipase activity assay and gut model suggest that the crude seaweed homogenate and ethanol supernatant extract for these particular seaweeds are potential pancreatic lipase inhibitors which have the potential to control dietary fat digestion. Furthermore, the physicochemical nature of the homogenate when in solution in the gut lumen provides capacity for binding of dietary fatty acids, a potential secondary mechanism for the prevention of fat digestion. Previous studies indicate that psyllium mucilloid dietary fiber products may reduce gastrointestinal side effects when taken concomitantly with Orlistat (Cavaliere et al. 2001). The seaweed homogenate may offer similar protection, alongside the polyphenol-rich supernatant. Human intervention studies would be required to evidence weight loss, confirm acceptability, and record any side effects of seaweed-enriched foodstuffs; however, this may be promising area for further study.
